# Dulaglutide treatment reverses depression‐like behavior and hippocampal metabolomic homeostasis in mice exposed to chronic mild stress

**DOI:** 10.1002/brb3.3448

**Published:** 2024-03-06

**Authors:** Man Jin, Shipan Zhang, Boya Huang, Litao Li, Hao Liang, Aihua Ni, Lina Han, Peng Liang, Jing Liu, Haishui Shi, Peiyuan Lv

**Affiliations:** ^1^ Department of Neurology Hebei Medical University Shijiazhuang China; ^2^ Department of Neurology Hebei General Hospital Shijiazhuang China; ^3^ Hebei Provincial Key Laboratory of Cerebral Networks and Cognitive Disorders Shijiazhuang China; ^4^ Neuroscience Research Center, Institute of Medical and Health Science Hebei Medical University Shijiazhuang China; ^5^ Cardiology Department Hebei General Hospital Shijiazhuang China; ^6^ Nursing School Hebei Medical University Shijiazhuang China

**Keywords:** depression, dulaglutide, liquid chromatography‐tandem mass spectrometry, metabolomics

## Abstract

**Introduction:**

Treatment strategies for depression based on interventions for glucose and lipid metabolism disorders are receiving increasing attention. Investigating the mechanism of their antidepressant effect and exploring new diagnostic and therapeutic biomarkers have attracted increasing attention. Dulaglutide, a long‐acting GLP‐1 receptor agonist, has been reported to alleviate cognitive deficits and neuronal damage. However, the antidepressant effect of dulaglutide and, especially, the underlying mechanism are still poorly understood. In this study, we aimed to explore the underlying biomarkers of depression and potential modulatory targets of dulaglutide in chronic mild stress (CMS) mice.

**Methods:**

Sixty mice were randomly divided into a control group (CON group), a CMS+Vehicle group (CMS+Veh group), a CMS+0.3 mg/kg dulaglutide group (Low Dula group), and a CMS+0.6 mg/kg dulaglutide group (High Dula group). Numerous behavioral tests, mainly the open field test, forced swimming test, and tail suspension test, were applied to evaluate the potential effect of dulaglutide treatment on anxiety‐ and depression‐like behaviors in mice exposed to chronic stress. Furthermore, a liquid chromatography–tandem mass spectrometry‐based metabolomics approach was utilized to investigate the associated mechanisms of dulaglutide treatment.

**Results:**

Three weeks of dulaglutide treatment significantly reversed depressive‐like but not anxiety‐like behaviors in mice exposed to chronic stress for 4 weeks. The results from the metabolomics analysis showed that a total of 20 differentially expressed metabolites were identified between the CON and CMS+Veh groups, and 46 metabolites were selected between the CMS+Veh and High Dula groups in the hippocampus of the mice. Comprehensive analysis indicated that lipid metabolism, amino acid metabolism, energy metabolism, and tryptophan metabolism were disrupted in model mice that experienced depression and underwent dulaglutide therapy.

**Conclusion:**

The antidepressant effects of dulaglutide in a CMS depression model were confirmed. We identified 64 different metabolites and four major pathways associated with metabolic pathophysiological processes. These primary data provide a new perspective for understanding the antidepressant‐like effects of dulaglutide and may facilitate the use of dulaglutide as a potential therapeutic strategy for depression.

## Graphical abstract

In this article, the antidepressant effects of dulaglutide in a CMS depression model were confirmed. We identified a total of 20 differentially metabolites between the CON and CMS+Veh groups and 46 metabolites between the CMS+Veh and High Dula groups in the hippocampus of mice. Four major pathways, lipid metabolism, amino acid metabolism, energy metabolism, and tryptophan metabolism, were ultimately determined to be associated with metabolic pathophysiological processes.

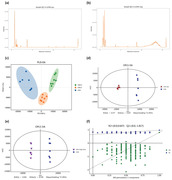

## 1 INTRODUCTION

As a chronic and recurrent mood disorder (Detka & Głombik, 2021), depression is a state of unhappiness and low mood, which is usually accompanied by an aversion to activity, low self‐esteem, fatigue, insomnia, weight loss, and so on (Wang et al., 2019; Zhou et al., 2020). According to the World Health Organization, depression affects more than 0.3 billion people worldwide, is one of the major causes of disability, and is a major health burden worldwide (WHO, 2017; Zhou et al., 2020). Currently, the widely available pharmacological treatments are synthetic chemical antidepressants, which require weeks to months to elicit a response, have high several side effects, and may even be toxic in high doses (Wang et al., 2019). As such, there is an urgent need for novel therapies with fewer adverse effects and greater efficacy.

A combination of genetic, neurological, and psychological factors contribute to the overall risk of depression (Zhang et al., 2022). A family history of depression, early life neglect and abuse, life stress, and comorbid physical illnesses all increase the risk of depression (Beurel E et al., 2020). Although the etiology of the disease has not been fully elucidated, chronic stress is regarded as one of the primary factors that predispose individuals to depression (Faria et al., 2014). Under chronic stress, overactivation of the sympathetic nervous system and dysregulation of the hypothalamic–pituitary–adrenal axis occur, leading to an increase in stress hormones such as catecholamines and glucocorticoids (Faria et al., 2014). The chronic mild stress (CMS) model is recognized as a reliable and practical rodent model of depression. As a result, it is broadly used to study the role of stress in the etiology of depression (Duan et al., 2022; Faria et al., 2014; Willner P., 2017). The hippocampus is a complex brain region involved in depression that exhibits morphological and functional alterations in response to stress (Zhang et al., 2022). Neuropathological findings have suggested a decrease in neuronal and glial size, loss of dendrites, reduced expression of synaptic markers, decreased neurogenesis, and an increase in apoptosis in the hippocampus of animal models of depression (Zhang et al., 2018; Zhang et al., 2022).

Recently, the metabolic aspects of depression have also been the focus of many studies. Moreover, several metabolic disorders, such as obesity and diabetes, are the main risk factors for depression (Detka & Głombik, 2021; Weina et al., 2018). Indeed, the successful application of antidiabetic agents in treating neurological diseases suggests that similar drugs may also have beneficial effects on depression (Weina et al., 2018). Glucagon‐like peptide‐1 (GLP‐1), which is secreted by intestinal L‐cells, is a peptide hormone that stimulates insulin secretion and inhibits the secretion of glucagon in the pancreas in a glucose‐dependent manner (An et al., 2022; Sharma et al., 2015). Worldwide, agonists of GLP‐1 receptors are broadly used in patients with type 2 diabetes (Sanada et al., 2021). In addition, GLP‐1 and its receptor agonists may have effects on sleep patterns, neuroprotective activities, anti‐inflammatory effects, and can even improve mental disorders, especially depression and cognition (An et al., 2022; Sharma et al., 2015; Weina et al., 2018). GLP‐1 analogs such as liraglutide have been proven to attenuate depressive‐ and anxiety‐like behavior in models of depression by improving hippocampal neuroplasticity (Sharma et al., 2015). Studies indicate that dulaglutide, a novel long‐acting GLP‐1 receptor agonist, may alleviate cognitive deficits and neuronal damage in rats with vascular dementia (Guan et al., 2023) and may ameliorate Alzheimer's disease‐like impairment of learning and memory ability induced by streptozotocin (Zhou et al., 2019). More importantly, a recent study in mice suggested that dulaglutide can prevent depression‐like behavior induced by chronic social defeat stress (CSDS) (Darwish et al., 2023). As dulaglutide is a new therapeutic candidate, data on the treatment of depression with dulaglutide are scarce, and the underlying mechanism of this effect has still not been sufficiently analyzed.

Metabolomic approaches are considered powerful tools for exploring the potential mechanisms of drugs containing multiple biochemical components (Wang et al., 2019). To date, hundreds of metabolomics investigations have been performed on metabolite alterations in animal models of depression, providing insight into its physiopathology (Pu et al., 2021). Liquid chromatography–tandem mass spectrometry (LC‐MS/MS) has been applied to identify metabolites in patients with depression and characterize the link between differential metabolites and cognitive neurological dysfunction (Duan et al., 2022). Although there has been evidence that GLP‐1 analogs such as liraglutide elicit metabolomic changes, such as changes in the levels of fatty acids and amino acids in the hypothalamus (Park et al., 2022), the metabolomic alterations evoked by dulaglutide in the hippocampus are poorly understood.

Based on the review of the literature, we aimed to use a metabolomics strategy to investigate the effect of dulaglutide in a CMS model in ICR mice and to identify potential biomarkers and related metabolic pathways.

## 2 MATERIALS AND METHODS

### 2.1 Animals

Adult male ICR mice (25.0−30.0 g, 7 weeks old) were purchased from Beijing Vital River Laboratory Animal Technology Co., Ltd. (Beijing, China). Mice had free access to food and water and were maintained under controlled conditions at 25°C with 55–65% humidity and a 12‐h light/dark schedule (lights on at 8:00 AM). Our protocol was approved by the Ethics Review Committee for Animal Experimentation of Hebei Medical University (Shijiazhuang, China) and was carried out in accordance with the National Institutes of Health Guide for the Care and Use of Laboratory Animals.

### 2.2 Experimental design

**FIGURE 1:**
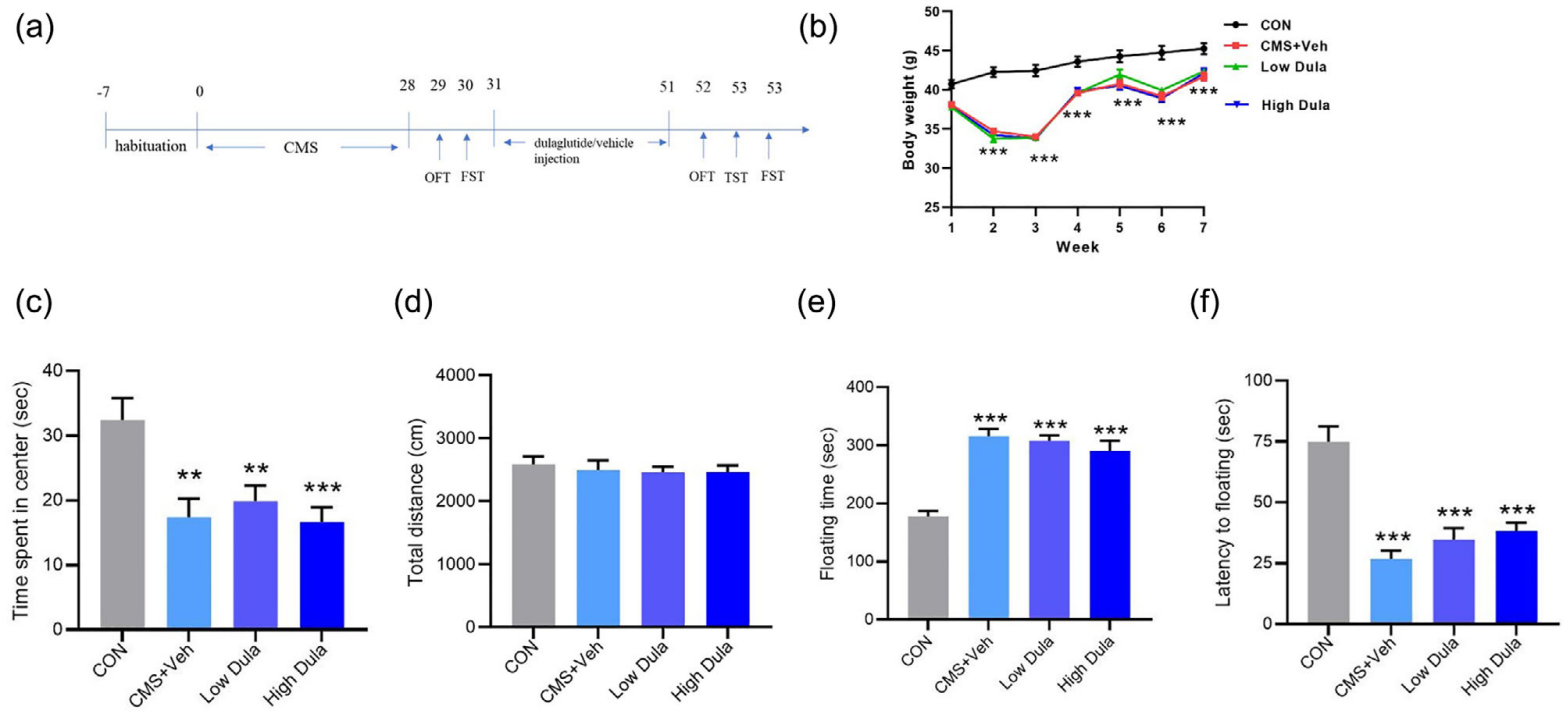
Chronic mild stress (CMS) model was successfully established. (a) Schematic of our study design. (b) Changes in body weight during the entire experimental period (*n* = 13–15). (c,d) Time spent in the center (c) and total distance (d) in the OFT after CMS handling (*n* = 13–15). (e,f) Floating time (e) and latency to floating (f) in the FST after CMS handling (*n* = 13–15). Data are presented as the mean ± SEM, least significant difference (LSD) post hoc test. *p < .05, **p < .01, ***p < .001 versus the CON group.

After acclimatization for 1 week, the 60 mice were randomly divided into four groups: the control group (CON), the CMS+Vehicle group (CMS+Veh), the CMS+0.3 mg/kg dulaglutide group (Low Dula), and the CMS+0.6 mg/kg dulaglutide group (High Dula). Mice in the CON group were not subjected to any stressors. The remaining three groups underwent CMS for 4 weeks. In addition, the Low Dula group and High Dula group were treated with an intraperitoneal injection of 0.3 or 0.6 mg/kg dulaglutide (diluted in saline, purchased from Eli Lilly, Japan), respectively, twice weekly for 3 weeks (Kimura et al., 2018). The CMS+Veh group received an intraperitoneal injection of Vehicle at the same dosage (Figure 1a).

### 2.3 CMS procedure

The CMS model of depression was established as previously described. Briefly, mice were randomly subjected to two to three different stressors for 28 continuous days: deprived of food for 12 h, deprived of water for 12 h, with a cage tilt for 24 h, with a pintail for 1 min, with a light/dark cycle reversal for 24 h, with wet bedding for 24 h, with crowded housing for 24 h, with empty bedding for 24 h, and with cold water for 5 min. The CON group was treated without any stress and was handled daily (Guo et al., 2022).

### 2.4 Behavioral tests

Body weight was measured weekly. Behavioral tests, including the open field test (OFT), tail suspension test (TST), and forced swimming test (FST), were conducted 24 h after the last stressor of the protocol or the last intraperitoneal injection of dulaglutide/vehicle. All behavioral tests were performed during the dark cycle by experimenters blinded to the groupings of the mice.

#### 2.4.1 OFT

Like in previous studies, mice were placed in the center of a square field (length 40 cm × width 40 cm × height 35 cm) under 20 lx intensity light. A video tracking system (SMART 3.0, Spain) was used to videotape the total activity of the mice for 5 min, during which time the mice were allowed to move freely. The data on the time spent in the central area and the total distance traveled were collected from the video (Zhang et al., 2022).

#### 2.4.2 FST

In a dark and quiet environment, the mice were individually placed in a plastic drum 10 cm in diameter and 35 cm in height containing approximately 20 cm of water (23−25°C) and were allowed to swim for 6 min. The floating time (the first 2 min) and latency to floating time (the last 4 min) were recorded manually. Floating was defined as the absence of any body movements other than those required to keep the head surfaced (Guo et al., 2022).

#### 2.4.3 TST

The TST was conducted according to the methods described in previous reports (Wang et al., 2023). The tail of each mouse was fixed with adhesive tape, and the mice were hung 40 cm above the floor for 6 min. The mice were separated from each other by a Styrofoam divider to avoid distraction. In the first 2 min, the latency to immobility was measured manually as the mice started to stay still. The immobility time, which was defined as the time spent resting on the limbs or body movements in the last 4 min, was manually recorded.

### 2.5 Sample collection and preparation

Twenty‐four hours after the final behavioral test, the mice were sacrificed. The brains were rapidly isolated on ice, and the hippocampus was dissected out. Then, the hippocampus was rapidly frozen in liquid nitrogen and stored at −80°C.

Twenty milligrams of sample were weighed into a 1.5‐mL EP tube, two small steel balls and 400 µL of methanol/water (4:1) were added, and the contents were ground and precooled at −40°C for 2 min. The whole samples were extracted by ultrasonication for 10 min in an ice‐water bath and stored at −20°C for 30 min. The supernatants (300 µL) were collected after centrifugation for 10 min (12,000 rpm, 4°C) and transferred to a new tube. The samples were resolubilized with 300 µL methanol/water (1:4), vortexed for 30 s, and sonicated for 3 min in an ice‐water bath. Then, 150 µL of the supernatant was obtained after centrifugation at 12,000 rpm for 10 min at 4°C; the mixture was filtered through 0.22 µm microfilters and transferred to LC vials (Wang et al., 2019). Quality control (QC) samples were prepared by mixing aliquots of extracts from all the samples.

### 2.6 LC‐MS/MS conditions

LC‐MS/MS analysis was performed on a Dionex Ultimate 3000 RS UHPLC fitted with a Q Exactive plus quadrupole‐orbitrap mass spectrometer equipped with a heated electrospray ionization (ESI) source (Thermo Fisher Scientific, Waltham, MA, USA). An ACQUITY UPLC HSS T3 column (1.8 µm, 2.1 × 100 mm) was used in both positive and negative modes. Water and acetonitrile/methanol (2/3, v/v), both containing 0.1% formic acid, were used as mobile phases A and B, respectively. The flow rate was set to 0.35 mL/min. The gradient conditions were set as follows: 0 min, 5% B; 2 min, 5% B; 4 min, 25% B; 8 min, 50% B; 10 min, 80% B; 14 min, 100% B; 15 min, 100% B; and 15.1 min, 5% and 16 min, 5% B. The injection volume was 5 µL. The column temperature was set at 45°C, and the sample temperature was 4°C. The mass range was from 100 *m*/*z* to 1200 *M*/*Z*. The resolution was set at 70,000 for the full MS scans and 17,500 for the MS/MS scans. The NCE/stepped NCE was set at 10, 20, and 40 eV. The mass spectrometer was operated as follows: spray voltage, 3800 V (+) and 3000 V (−); sheath gas flow rate, 50 arbitrary units; auxiliary gas flow rate, 8 arbitrary units; capillary temperature, 320°C; aux gas heater temperature, 350°C; and S‐lens RF level, 50.

For metabolomics analysis, the raw LC‐MS/MS data were processed by Progenesis QI V2.3 software (Nonlinear, Dynamics, Newcastle, UK), which was used for baseline filtering, peak identification, retention time correction, integration, peak alignment, and normalization. The main parameters used were a 5 ppm precursor tolerance, a 10 ppm product tolerance, and a 5% product ion threshold. The peaks with a missing value (ion intensity = 0) in more than 50% of the groups were removed, and the zero value was replaced by half of the minimum value. Compounds with a result score of less than 36 (out of 60) points were also removed. Potential biomarkers were identified by comparing the precise mass‐to‐charge ratio (*M*/*Z*), secondary fragments, and isotopic distribution based on The Human Metabolome Database, Lipidmaps (V2.3), Metlin, EMDB, PMDB, and self‐built databases.

A data matrix with positive and negative ion data was combined and imported into R to perform principal component analysis to observe the overall distribution between samples and the stability of the entire analysis process. Orthogonal partial least‐squares‐discriminant analysis (OPLS‐DA) and partial least‐squares‐discriminant analysis (PLS‐DA) were applied to distinguish the differentially abundant metabolites between groups. Variable importance of projection (VIP) values obtained from the OPLS‐DA model (VIP > 1.0) and two‐tailed Student's *t*‐test (*p* < .05) were considered to indicate statistical significance (Fan et al., 2021).

### 2.7 Statistical analysis

The data from the behavioral tests are presented as the means ± standard errors of the means (SEMs) and were statistically analyzed using SPSS 25.0 software (IBM, USA). One‐way ANOVA followed by the least significant difference (LSD) post hoc test was used for multiple‐group comparisons. *p* < .05 was regarded as statistically significant.

## 3 RESULTS

### 3.1 Dulaglutide reverses depressive‐like behavior in stressed mice

**FIGURE 2:**
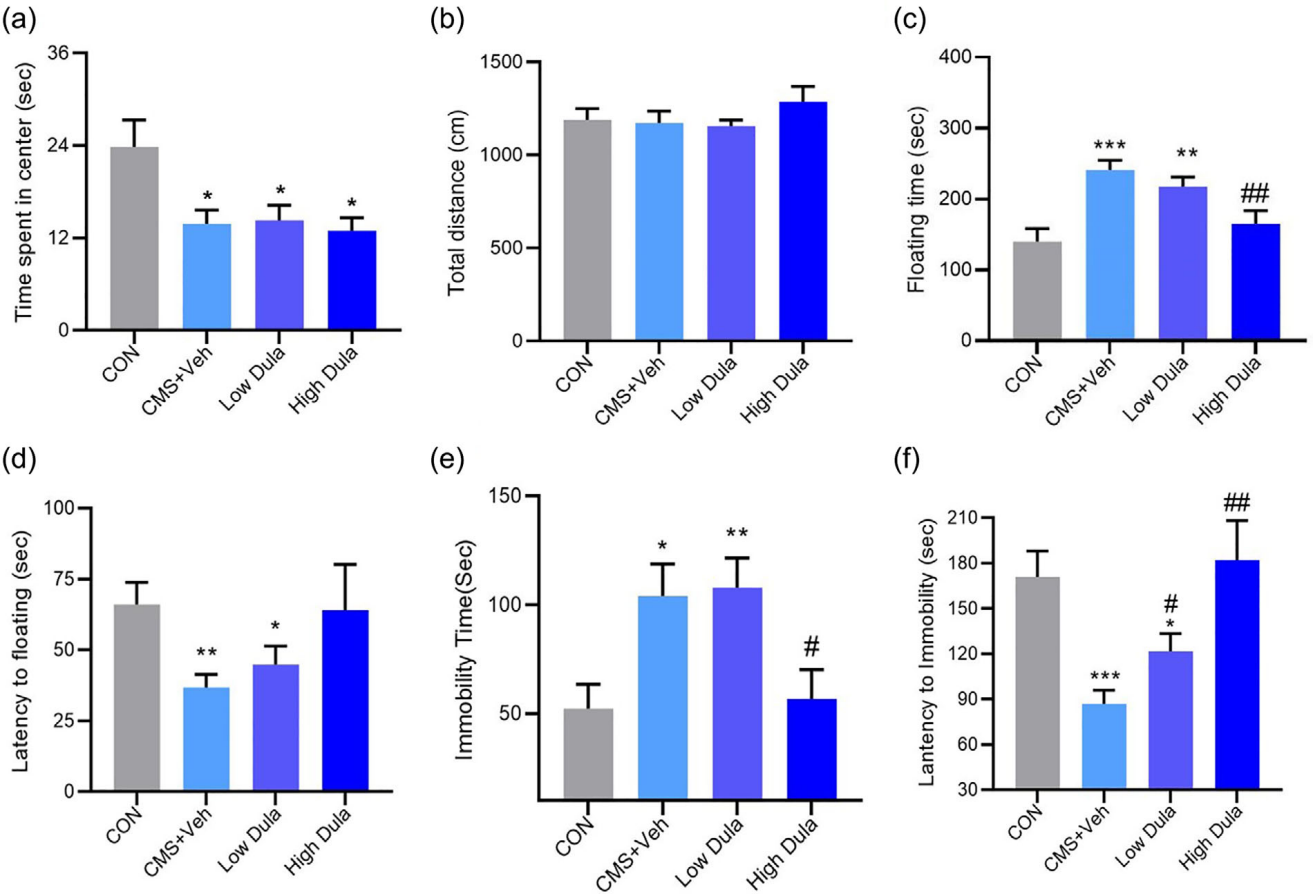
Dulaglutide reverses depressive‐like behavior in stressed mice. (a,b) Time spent in the center (a) and total distance (b) in the open field test (OFT) after dulaglutide treatment (*n* = 13–15). (c,d) Floating time (c) and latency to floating (d) in the forced swimming test (FST) after dulaglutide treatment (*n* = 13–15). (e,f) Immobility time (e) and latency to immobility (f) in the tail suspension test (TST) after dulaglutide treatment (*n* = 13–15). Data were presented as the mean ± SEM, least significant difference (LSD) post hoc test, *p < .05, **p < .01, ***p < .001 versus the CON group. ^#^ *p* < .05, ^##^ *p* < .01, ^###^ *p* < .001 versus the CMS+Veh group.

The mice subjected to 4 weeks of CMS eventually manifested depressive‐ and anxiety‐like behavior. In the OFT, CMS resulted in a shorter time spent in the central zone (*F* (3,14) = 6.497, *p* < .05, Figure 1c,d) but no difference in total distance. Compared to the CON group, the CMS mice manifested a significant decrease in body weight (all *p* < .05, *n* = 13–15, Figure 1b) and spent a longer time immobile (*F* (3,12) = 24.214, *p* < .05, Figure 1e,f) during the FST, indicating a loss of appetite and increased desperation. These results indicated the successful establishment of the CMS model. Nevertheless, the interventions of dulaglutide significantly alleviated depressive‐like behavior, which demonstrated that mice in the high Dula group displayed a longer struggling time in the FST (*F* (3,13) = 8.146, *p* < .05, Figure 2c,d) and a shorter immobility time in the TST (*F* (3,12) = 4.400, *p* < .05, Figure 2e,f), similar to that in the CON group. However, dulaglutide did not improve anxiety‐like behavior in mice (*F* (3,13) = 4.713, *p* > .05, Figure 2a,b).

### 3.2 LC‐MS/MS chromatogram of hippocampal tissue samples

**FIGURE 3:**
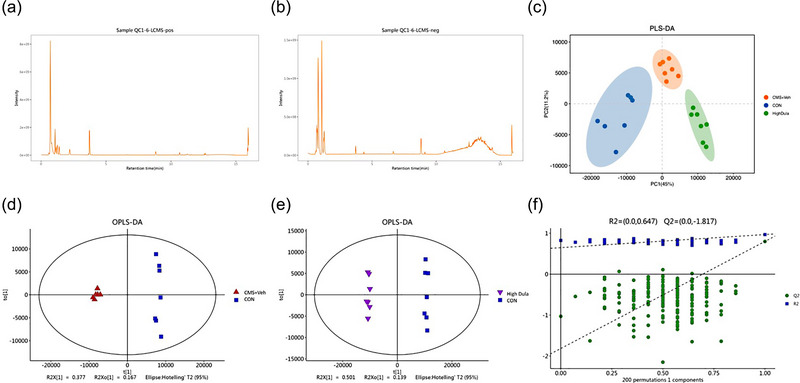
Multivariate statistical analysis between CON, CMS + Veh, and High Dula groups. (a,b) Superimposed chromatograms of quality control (QC) samples in positive and negative modes (*n* = 6). (c) Partial least squares‐discriminate analysis (PLS‐DA) scores plot. (d,e) Orthogonal partial least‐squares‐discriminant analysis (OPLS‐DA) scores plot. (f) Statistical validation of the PLS‐DA model through 200× permutation testing. CMS, chronic mild stress; CON, control; CMS+Veh, CMS+Vehicle; High Dula, CMS+0.6 mg/kg dulaglutide.

We collected hippocampal tissues from each group of mice for nontargeted metabolic studies to identify the metabolites that were altered in the brains of CMS‐induced depressed mice and to ascertain the effect of dulaglutide on mice with depression. The QC sample was run every 10 samples in the analysis to validate the stability of the LC‐MS platform, and total ion current chromatograms of the metabolites were recorded. Figures 3a and 3b show chromatograms in positive mode and negative mode, respectively, suggesting that the system for LC‐MS was reproducible and satisfactory. Multivariate analysis was subsequently conducted based on the signals detected in the hippocampal samples of the CON, CMS + Veh, and High Dula groups in the ESI+ and ESI− directions.

### 3.3 Identification of metabolite biomarkers discriminating the CMS and control groups

A clear separation of metabolites was observed by PLS‐DA analysis in the CON, CMS + Veh, and High Dula groups (Figure 3c). The model parameters of PLS‐DA were R2X = 0.699, R2Y = 0.967, and Q2 = 0.799.

To maximize the discrimination, we also established an OPLS‐DA model (Figure 3d,e), which revealed that the three groups were separated from each other. For the CON and CMS + Veh groups, the parameters of the loading plots were R2X = 0.623, R2Y = 0.995, and Q2 = 0.938, for the CMS+Veh and High Dula groups, these values were: R2X = 0.547, R2Y = 0.999, and Q2 = 0.915; and for the CON and High Dula group, those parameters were: R2X = 0.695, R2Y = 0.999, and Q2 = 0.973. To prevent the OPLS‐DA model from overfitting, sevenfold cross validation and 200‐response permutation testing were performed (Figure 3f). A value of the above parameters approaching 1.0 indicated that they were reliable and had good predictive ability.

### 3.4 Identification of potential biomarkers

**FIGURE 4:**
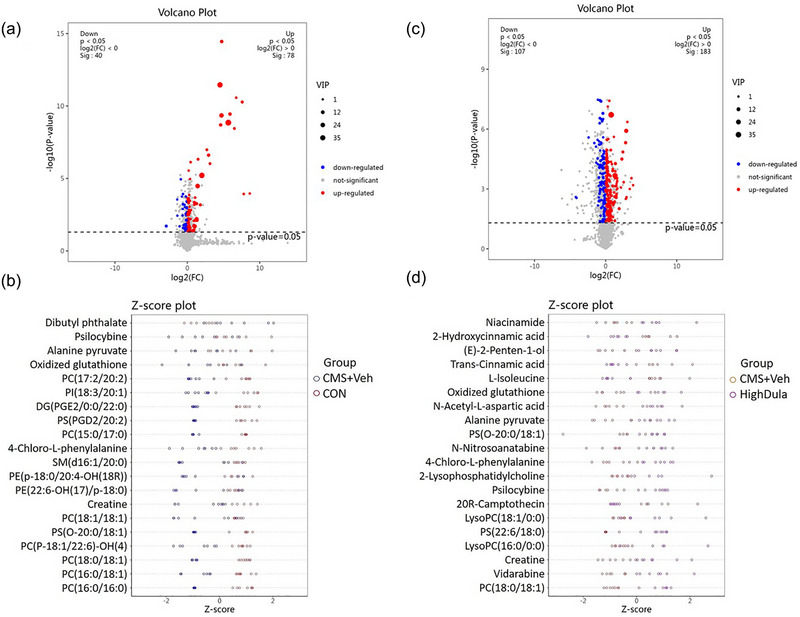
Different metabolites in CON, CMS+Veh, and High Dula groups. (a,c) The different metabolites between CON/CMS+Veh group (a) and High Dula/CMS+Veh group (c) are shown by the volcano plot. Red indicates significantly up‐regulated metabolites, whereas blue indicates significantly down‐regulated metabolites after dulaglutide treatment. (b,d) Z‐Score analysis of significantly different metabolites by Top 20 VIP ranking for both CON/CMS+Veh group (b) and High Dula/CMS+Veh group (d). CMS, chronic mild stress; CON, control; CMS+Veh, CMS+Vehicle; High Dula, CMS+0.6 mg/kg dulaglutide; VIP, variable importance of projection.

**FIGURE 5:**
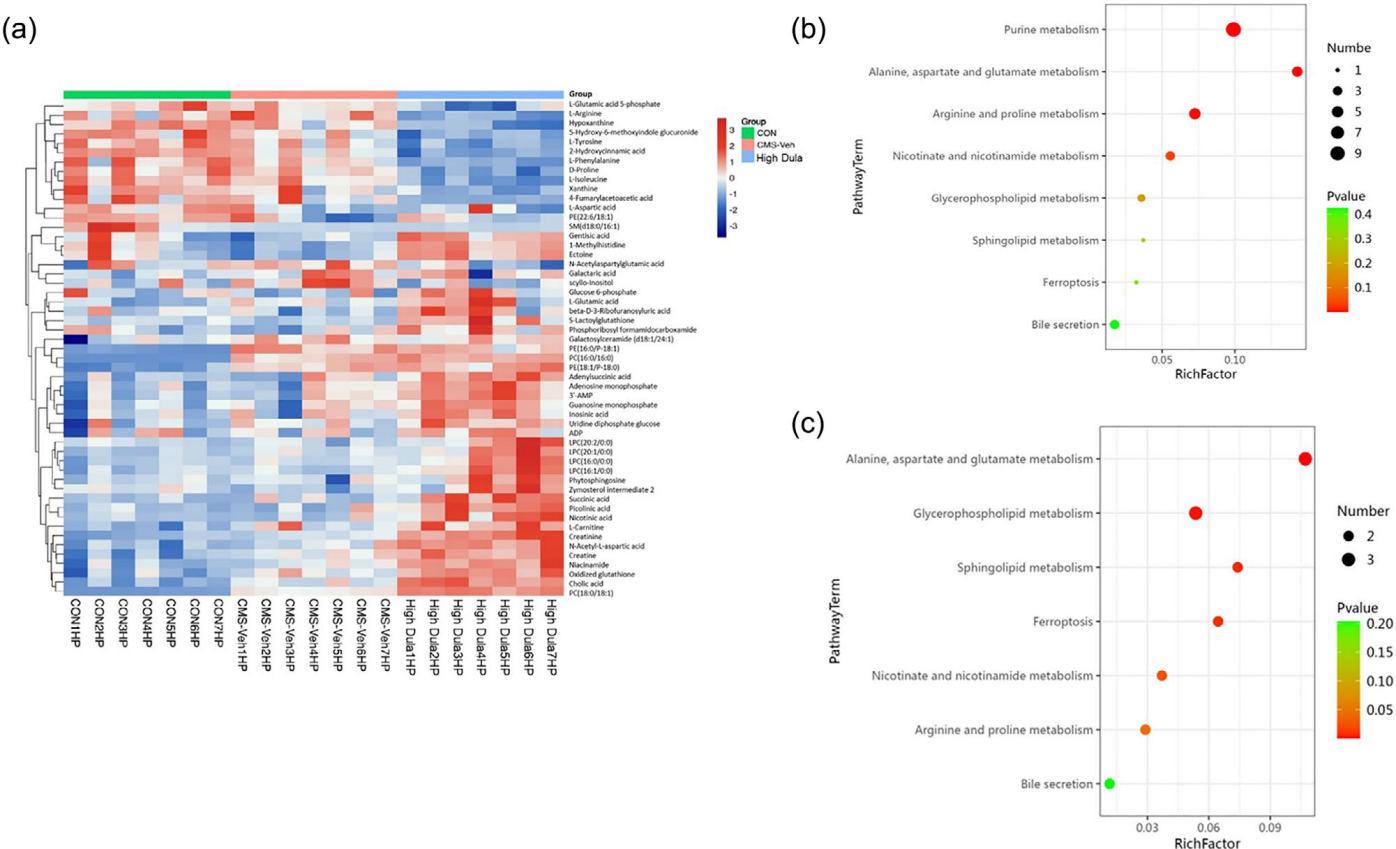
Heatmap and metabolic pathway analysis in CON, CMS+Veh, and High Dula groups. (a) The different metabolites are shown by heatmap. Each column is a sample, each row is a metabolite, the color reflects the relative abundance of metabolites, and similar samples are clustered together. (b,c) Bubble chart of the metabolic pathway analysis of CON/CMS+Veh (b) and CMS+Veh/High Dula group (c). The *p*‐value and impact factor are represented by dot color and dot size, respectively. CMS, chronic mild stress; CON, control; CMS+Veh, CMS+Vehicle; High Dula, CMS+0.6 mg/kg dulaglutide.

OPLS‐DA mode was also used to evaluate the differential metabolites that were identified with VIP > 1 and *p* < .05 in samples. In total, 20 endogenous metabolites were identified as potential biomarkers in the CON and CMS+Veh groups (Table 1), and 46 different metabolites were selected in the CMS+Veh and High Dula groups (Table 2; Figures 4a–d and 5a). We analyzed the significantly altered canonical pathways based on public databases and focused on the following pathways: lipid metabolism, amino acid metabolism, energy metabolism, and tryptophan metabolism. The detailed results of the pathway analyses are shown in Figure 5b,c.

**TABLE 1:** Differentially abundant metabolites in the hippocampus between the CON and CMS+Veh groups.

Metabolite	RT (min)	Ion mode	Metabolic pathway	Log2 (FC)	Trend
PC (18:0/18:1)	12.58	ESI+	Glycerophosphocholines	4.51	↑**
PC (16:0/16:0)	12.58	ESI+	Glycerophosphocholines	5.65	↑**
Creatine	0.70	ESI+	Amino acids	0.11	↑**
Oxidized glutathione	1.08	ESI+	Amino acids	0.24	↑*
SM (d18:0/16:1)	11.34	ESI+	Phosphosphingolipids	−2.91	↓*
PE (22:6/18:1)	10.55	ESI‐	Glycerophosphoethanolamines	−0.18	↓*
PE (16:0/P‐18:1)	12.23	ESI+	Glycerophosphoethanolamines	2.69	↑**
PE (18:1/P‐18:0)	12.30	ESI+	Glycerophosphoethanolamines	1.48	↑**
N‐Acetyl‐L‐aspartic acid	1.08	ESI+	Amino acids	0.13	↑*
2‐Hydroxycinnamic acid	1.12	ESI+	Hydroxycinnamic acids and derivatives	−0.17	↓**
Niacinamide	1.08	ESI+	Pyridinecarboxylic acids and derivatives	0.08	↑*
L‐Carnitine	0.70	ESI+	Quaternary ammonium salts	0.17	↑*
L‐Glutamic acid	0.70	ESI‐	Amino acids	0.094	↑*
Zymosterol intermediate 2	13.16	ESI+	Cholestane steroids	−0.23	↓*
Cholic acid	8.85	ESI+	Bile acids	0.092	↑**
L‐Aspartic acid	0.69	ESI‐	Amino acids	−0.11	↓*
Galactosylceramide (d18:1/24:1)	14.83	ESI+	Glycosphingolipids	0.51	↑*
scyllo‐Inositol	0.67	ESI‐	Alcohols and polyols	0.090	↑*
LPC(20:1/0:0)	11.77	ESI+	Glycerophosphocholines	0.33	↑*
Galactaric acid	0.70	ESI‐	Carbohydrates and carbohydrate conjugates	0.14	↑*

Abbreviations: ↓, downregulation;↑, upregulation; Con, control group; RT, retention time; FC, fold change; PC, phosphatidylcholine; PE, phosphatidylethanolamines; LPC, lysophosphatidylcholine.

*p < .05, **p < .01.

**TABLE 2:** Differentially abundant metabolites in the hippocampus between the High Dula and CMS+Veh groups.

Metabolite	RT (min)	Ion mode	Metabolic pathway	Log2 (FC)	Trend
PC (18:0/18:1)	12.58	ESI+	Glycerophosphocholines	0.77	↑**
Creatine	0.70	ESI+	Amino acids, peptides, and analogues	0.10	↑**
LPC (16:1/0:0)	10.09	ESI+	Glycerophosphocholines	0.57	↑**
LPC (16:0/0:0)	10.67	ESI+	Glycerophosphocholines	0.50	↑*
PC (18:0/18:1)	11.117	ESI+	Glycerophosphocholines	0.77	↑*
LPC (20:1/0:0)	11.77	ESI+	Glycerophosphocholines	0.71	↑*
LPC (20:3/0:0)	10.69	ESI+	Glycerophosphocholines	0.81	↑*
LPC (0:0/18:1)	10.89	ESI‐	Glycerophosphocholines	0.59	↑*
N‐Acetyl‐L‐aspartic acid	1.087	ESI+	Amino acids, peptides, and analogues	0.24	↑**
Oxidized glutathione	1.087	ESI+	Amino acids, peptides, and analogues	0.22	↑**
L‐Isoleucine	1.407	ESI+	Amino acids, peptides, and analogues	−0.30	↓**
2‐Hydroxycinnamic acid	1.127	ESI+	Hydroxycinnamic acids and derivatives	−0.37	↓**
Niacinamide	1.087	ESI+	Pyridinecarboxylic acids and derivatives	0.14	↑**
3′‐AMP	0.747	ESI+	Unclassified	0.49	↑**
Zymosterol intermediate 2	13.16	ESI+	Cholestane steroids	0.60	↑*
N‐Acetylaspartylglutamic acid	1.15	ESI+	Amino acids, peptides, and analogues	−0.26	↓*
L‐Arginine	0.62	ESI+	Amino acids, peptides, and analogues	−0.47	↓**
Inosinic acid	0.76	ESI‐	Purine ribonucleotides	0.57	↑*
S‐Lactoylglutathione	1.32	ESI+	Amino acids, peptides, and analogues	1.91	↑*
Cholic acid	8.84	ESI+	Bile acids, alcohols and derivatives	0.14	↑**
D‐Proline	0.73	ESI+	Amino acids, peptides, and analogues	−0.24	↓**
Adenosine monophosphate	0.75	ESI‐	Purine ribonucleotides	0.52	↑**
L‐Carnitine	0.70	ESI+	Quaternary ammonium salts	0.14	↑*
Adenylsuccinic acid	1.29	ESI+	Biphenyls and derivatives	0.63	↑*
Glucose 6‐phosphate	0.76	ESI‐	Carbohydrates and carbohydrate conjugates	0.25	↑*
Guanosine monophosphate	0.75	ESI‐	Purine ribonucleotides	0.40	↑*
scyllo‐Inositol	0.67	ESI‐	Alcohols and polyols	−0.12	↓**
Succinic acid	1.25	ESI‐	Dicarboxylic acids and derivatives	1.24	↑**
Nicotinic acid	1.08	ESI+	Pyridinecarboxylic acids and derivatives	1.52	↑**
Ectoine	13.77	ESI+	Amino acids, peptides, and analogs	0.16	↑**
Hypoxanthine	1.09	ESI‐	Purines and purine derivatives	−0.22	↓**
4‐Fumarylacetoacetic acid	1.159	ESI‐	Medium‐chain keto acids and derivatives	−4.10	↓**
Picolinic acid	0.79	ESI+	Pyridinecarboxylic acids and derivatives	1.19	↑**
1‐Methylhistidine	13.77	ESI+	Amino acids, peptides, and analogs	0.15	↑**
Xanthine	1.09	ESI‐	Purines and purine derivatives	−0.27	↓*
5‐Hydroxy‐6‐methoxyindole glucuronide	1.13	ESI+	Carbohydrates and carbohydrate conjugates	−0.34	↓**
Phytosphingosine	9.75	ESI+	Amines	0.46	↑*
Phosphoribosyl formamidocarboxamide	0.80	ESI+	1‐Ribosyl‐imidazolecarboxamides	0.24	↑*
L‐Tyrosine	1.14	ESI‐	Amino acids, peptides, and analogs	−0.49	↓**
ADP	0.84	ESI‐	Purine ribonucleotides	0.18	↑*
beta‐D‐3‐Ribofuranosyluric acid	0.91	ESI‐	Purines and purine derivatives	0.30	↑*
Uridine diphosphate glucose	0.64	ESI‐	Pyrimidine nucleotide sugars	0.11	↑*
L‐Phenylalanine	2.21	ESI‐	Amino acids, peptides, and analogs	−0.31	↓**
Creatinine	12.58	ESI+	Amino acids, peptides, and analogs	1.16	↑**
Gentisic acid	13.77	ESI+	Benzoic acids and derivatives	0.12	↑**
L‐Glutamic acid 5‐phosphate	0.86	ESI‐	Amino acids, peptides, and analogs	−0.29	↓**

Abbreviations: ↓, downregulation;↑, upregulation; RT, retention time; FC, fold change; PC, phosphatidylcholine; LPC, lysophosphatidylcholine.

*p < .05, **p < .01.

## 4 DISCUSSION

The CMS model is a reliable and practical experimental model of depression that is broadly used in the investigation of depression mechanisms (Antoniuk et al., 2019). Immobility time in the TST and FST, which reflects the degree of desperation in the mice (Sasibhushana et al., 2019), are important indices for evaluating antidepressant‐like effects (Duan et al., 2022). To our knowledge, our study is the first to investigate the antidepressant effect of dulaglutide in a CMS‐induced depression model. Interestingly, we noted that although the mice exhibited significantly depressed behavior after 4 weeks of CMS, these depressive‐like behaviors were reversed after treatment with dulaglutide, as indicated by a marked decrease in immobility time in the FST and TST.

These results are similar to the findings of Darwish et al. Their prior work revealed that dulaglutide reversed the CSDS‐induced behavioral changes observed in the social interaction test, elevated plus‐maze test, and sucrose preference test (Darwish et al., 2023). These authors further showed that dulaglutide plays a neuroprotective role by improving the cAMP/PKA pathway to inhibit NLRP3 inflammasome‐induced proinflammatory cytokines (Darwish et al., 2023). As an essential addition, we conducted an LC‐MS/MS metabolomics study combined with multivariate statistical analysis in our work.

Metabolomics can be used as a comprehensive examination of metabolic disorders occurring in the body, through which we can search for potential pathophysiological mechanisms and assess the efficacy of drugs (Rinschen et al., 2019). We observed a clear separation of the CON group, CMS+Veh group, and High Dula group, which revealed that metabolic disorders induced by chronic stress were notably modulated by dulaglutide treatment. A total of 20 and 46 potential biomarkers were identified among normal/CMS mice and CMS/dulaglutide‐treated mice, respectively, which were involved in glycerophospholipid metabolism; purine metabolism; arginine and proline metabolism; ferroptosis, alanine, aspartate, and glutamate metabolism; nicotinate and nicotinamide metabolism; sphingolipid metabolism; and bile secretion. These metabolic pathways were distributed into four main categories according to their biochemical functions to further comprehend the potential mechanisms of depression. The main pathways were involved in: (1) lipid metabolism (glycerophospholipid metabolism, sphingolipid metabolism); (2) amino acid metabolism (arginine and proline metabolism; alanine, aspartate, and glutamate metabolism); (3) energy metabolism (purine metabolism); and (4) other metabolic pathways (nicotinate and nicotinamide metabolism). The following pathways correlated with these metabolic pathways and depression.

### 4.1 Lipid metabolism

We found that lipid metabolism pathways were the most likely potential targets through which dulaglutide affects depression. Sphingolipids, lysophosphatidylcholine (LPC), lysophosphatidylethanolamine (LPE), phosphatidylcholine (PC), phosphatidylethanolamine (PE), and phosphatidylinositol (PI) are all involved in the pathological changes observed in depression and the therapeutic effect of dulaglutide. Consistent with the findings of previous research (Faria et al., 2014), in the CMS+Veh group, galactosylceramide and SM (d18:0/16:1) underwent metabolic perturbation, and the levels of LPC (20:1/0:0), PC (18:0/18:1), PC (16:0/16:0), PE (16:0/P‐18:1), and PE (18:1/P‐18:0) were significantly increased, which indicated that depression could cause the perturbation of glycerophospholipid metabolism and sphingolipid metabolism.

Both galactosylceramide and SM (d18:0/16:1) are important intermediates in sphingolipid metabolism. Sphingolipids may induce synaptic abnormalities that lead to neurological disorders (Wang et al., 2019). Glycerophospholipids, such as PCs, PIs, PEs, and lysophospholipids (LPCs, LPEs, LPIs), are critical for many biological processes (Geng et al., 2019). They are the main components of nerve cell membranes and myelin, especially in the prefrontal cortex and hippocampus regions, and have been shown to regulate depression in the brain (Hu et al., 2022). A study examining healthy controls and MDD patients showed that PCs and PEs were strongly associated with the severity of depression (Liu et al., 2016). Studies have also shown that PCs and PEs are upregulated in the brains of depressed mice (Gao et al., 2021; Geng et al., 2019). The increase in PCs may suggest a protective response to stress (Faria et al., 2014). LPC (16:1/0:0), LPC (16:0/0:0), LPC (20:2/0:0), LPC (20:1/0:0), LPC (20:3/0:0), LPC (0:0/18:1), and phytosphingosine were upregulated after dulaglutide treatment. Huang et al. showed that CMS disturbs glycerophospholipid metabolism; however, glycerophospholipid metabolism could be regulated by ellagic acid treatment to exert an antidepressant effect (Huang et al., 2020). In line with these findings, our results also suggested that there are important associations between lipid metabolism and the antidepressant effect of dulaglutide.

### 4.2 Amino acid metabolism disorder

Consistent with the findings of a previous meta‐analysis (Pu et al., 2021), the downregulation of N‐acetyl‐L‐aspartic acid (NAA) in the CMS model group was found in our study. NAA is one of the richest metabolites in the vertebrate nervous system (Kelley & Stamas, 1992). Aspartic acid is synthesized in the mitochondria and is regarded as a marker of neuronal density, and its metabolism is the main pathway among brain regions (Fan et al., 2021). Studies have indicated that NAA levels are decreased in the amygdala in a model of CSDS and chronic unpredictable mild stress in rats (Fan et al., 2021). In our study, its level was upregulated in the hippocampus after dulaglutide therapy, indicating that it may be a potential therapeutic target for depression.

We noticed that L‐glutamic acid and L‐arginine were upregulated in the CMS model group. The perturbation of glutamate metabolism in the pathology of depression is controversial. L‐glutamic acid acts as an amino acid neurotransmitter and contributes to changes in cortical excitability and inhibition when its expression is abnormal in the brains of individuals with affective disorders (Du et al., 2021). A meta‐analysis of 241 animal metabolomics studies on depression revealed decreased levels of glutamic acid, arginine, and proline in the brain (Pu et al., 2021). However, some rodent experiments have demonstrated that acute stress can increase glutamate levels in the hippocampus and amygdala (Reznikov et al., 2007). Clinical studies have also shown that a higher level of glutamic acid is present in patients with mental illness (Altamura et al., 1993). This finding is consistent with the results of our study. L‐arginine is considered a conditionally essential amino acid and is synthesized from proline, glutamine, and glutamate in humans and other mammals via the intestinal–renal axis (Huang et al., 2020). In the CMS+Veh group, an upregulation of L‐arginine was observed, which could, however, be regulated by intervention with dulaglutide, while causing a decrease in proline levels. Arginine–proline metabolism is connected with nitric oxide cycling (Cunha et al., 2018，Morris.,2006). Arginine, as the precursor of proline, is converted to nitric oxide and citrulline with the help of nitric oxide synthases (Du et al., 2021). The decrease in arginine and proline caused by dulaglutide, thus, may have indirectly decreased NO production to exert a neuroprotective effect.

### 4.3 Energy metabolism

Energy deficiency plays a major role in the development of depression (Wang et al., 2019). Preclinical studies have noted that supplementation with creatine or ATP leads to antidepressant activity (Cao et al., 2013; Cunha et al., 2018). Consequently, energy metabolism may act as a prospective target for the treatment of depression (Pu et al., 2021). In the present study, dulaglutide treatment induced energy metabolism perturbation. Increases in succinic acid, creatine, gentisic acid, ADP, guanosine monophosphate, and adenosine monophosphate (An et al., 2022), which are involved in recycling ATP, were observed after dulaglutide intervention. ATP is considered a neurotransmitter and neuromodulator in the central nervous system (Pedrazza et al., 2008). Adenosine and guanosine, products of ATP catabolism, have been proven to modulate cognitive function and are related to affective disorders, such as anxiety and depression (Pedrazza et al., 2008; Yu et al., 2017). The anti‐depressive effects of guanosine have been revealed. Since it could re‐establish the normal activity of endogenous antioxidant enzymes to attenuate oxidative stress in the hippocampus, it also has the ability to mediate nitric oxide‐cGMP, NMDA receptors, and the PI3K/mTOR pathway (Yu et al., 2017).

### 4.4 Other metabolic pathways

In addition, dulaglutide increased the levels of niacinamide and picolinic acid (PIC), indicating its therapeutic effects. Tryptophan (TRP), as a precursor for the synthesis of 5‐hydroxytryptamine, nicotinamide adenine dinucleotide (NAD), and nicotinic acid, can be transported into the brain through the blood and plays an essential role in depression (Wang et al., 2019). Nicotinamide has antidepressant effects in combination with TRP through the inhibition of peripheral breakdown (Weina et al., 2018). Recent studies have demonstrated that niacinamide supplementation can improve depressive mood in young adults with severe subclinical depression in the short term (Tsujita et al., 2019). Ninety‐five percent of TRP is catabolized via the kynurenine pathway, eventually leading to the formation of neurotoxic quinolinic acid or neuroprotective PIC (Lovelace et al., 2017). Increasing PIC levels was verified to be a beneficial therapeutic strategy in patients with bipolar disorder. In addition, individuals with low PIC may be vulnerable to suicidal ideation (Trepci et al., 2021).

In conclusion, for the first time, we confirmed the antidepressant effects of dulaglutide in a CMS depression model by performing certain behavioral tests. An LC‐MS/MS‐based hippocampal metabolomics approach was established and coupled with multivariate statistical analysis to explore the underlying mechanism involved. The potential diagnostic biomarkers of depression and the antidepressant effect of dulaglutide were intimately linked to the mediation of several metabolic pathways, mainly those involving energy metabolism, amino acid metabolism, and lipid metabolism, in the present study. Here it must be added that, from a clinical standpoint, the unpredictable stress‐induced model we used here does not simulate the type of depression and lacks an estimate of the side consequences of the drug; therefore, its safety in nondiabetic patients cannot be entirely affirmed. On the other hand, metabolomic analyses reflect only a collective “snapshot” of metabolic disorders, which may involve a variety of confounding factors and have substantial individual variation (Weina et al., 2018). Moreover, we measured only the hippocampus, and other regions associated with depression were not examined. Despite these limitations, the identified metabolites are potential targets for further laboratory investigations, and dulaglutide is a potential antidepressant treatment.

## AUTHOR CONTRIBUTIONS

**Man Jin**: Investigation; data curation; methodology; validation; formal analysis; writing—original draft. **Shipan Zhang**: data curation; methodology; validation. **Boya Huang**: data curation; methodology. **Litao Li**: Investigation; validation. **Hao Liang**: Investigation; methodology. **Aihua Ni**: Supervision. **Lina Han**: Validation. **Peng Liang**: Methodology. **Jing Liu**: Investigation. **Haishui Shi**: Conceptualization; supervision; validation; methodology. **Peiyuan Lv**: Conceptualization; writing—review and editing; supervision; project administration.

## CONFLICT OF INTEREST STATEMENT

The authors declare no conflicts of interest.

### PEER REVIEW

The peer review history for this article is available at https://publons.com/publon/10.1002/brb3.3448.

## Data Availability

The data that support the findings of this study are available from the corresponding author upon reasonable request.
